# Three-dimensional flat Landau levels in an inhomogeneous acoustic crystal

**DOI:** 10.1038/s41467-024-46517-z

**Published:** 2024-03-11

**Authors:** Zheyu Cheng, Yi-Jun Guan, Haoran Xue, Yong Ge, Ding Jia, Yang Long, Shou-Qi Yuan, Hong-Xiang Sun, Yidong Chong, Baile Zhang

**Affiliations:** 1 Division of Physics and Applied Physics, School of Physical and Mathematical Sciences, Nanyang Technological University, Singapore, 637371 Singapore; 2 Research Center of Fluid Machinery Engineering and Technology, School of Physics and Electronic Engineering, Jiangsu University, 212013 Zhenjiang, China; 3 State Key Laboratory of Acoustics, Institute of Acoustics, Chinese Academy of Sciences, 100190 Beijing, China; 4 Department of Physics, The Chinese University of Hong Kong, Shatin, Hong Kong SAR China; 5 Centre for Disruptive Photonic Technologies, Nanyang Technological University, Singapore, 637371 Singapore

**Keywords:** Topological matter, Acoustics

## Abstract

When electrons moving in two dimensions (2D) are subjected to a strong uniform magnetic field, they form flat bands called Landau levels (LLs). LLs can also arise from pseudomagnetic fields (PMFs) induced by lattice distortions. In three-dimensional (3D) systems, there has been no experimental demonstration of LLs  as a type of flat band thus far. Here, we report the experimental realization of a flat 3D LL in an acoustic crystal. Starting from a lattice whose bandstructure exhibits a nodal ring, we design an inhomogeneous distortion corresponding to a specific pseudomagnetic vector potential (PVP). This distortion causes the nodal ring states to break up into LLs, including a zeroth LL that is flat along all three directions. These findings suggest the possibility of using nodal ring materials to generate 3D flat bands, allowing access to strong interactions and other attractive physical regimes in 3D.

## Abstract (web summary)

Artificial magnetic fields have been meticulously engineered in a 3D acoustic crystal, facilitating the creation of 3D flat bands through Landau quantization of quasiparticles arising from nodal-ring band degeneracies.

## Introduction

LLs first arose in Landau’s 1930 derivation of the magnetic susceptibility of metals, based on a quantum mechanical model of nonrelativistic electrons in a uniform magnetic field^1^. If the electrons are constrained to the 2D plane perpendicular to the magnetic field, the LLs form a set of equally spaced flat energy bands independent of the in-plane momentum. Such 2D LLs were subsequently found to exhibit quantized Hall conductance (the integer quantum Hall effect) due to their nontrivial band topology^2,3^. Other 2D models host different types of LLs; for example, particles governed by a 2D Dirac equation (such as electrons near the Dirac points of graphene), when subjected to a uniform magnetic field, produce 2D LLs that are flat but unequally spaced in energy, with a zeroth LL at zero energy^4,5^. Flat bands such as 2D LLs are of broad interest in multiple fields of physics since their high density of states provides access to strong-interaction regimes^6–11^, such as strong inter-electron interactions in condensed matter systems, which give rise to phenomena like the fractional quantum Hall effect^6,7,12,13^, and strong light-matter coupling in optoelectronic systems^14,15^. Although LLs are not the only way to achieve flat bands, they are attractive because of their rich physics and relative accessibility. Aside from using real magnetic fields, LLs can also arise from PMFs induced by lattice distortions without breaking time-reversal invariance^16^. This is achievable in electronic materials through strain engineering^16–19^ or inter-layer twisting^13^, and in synthetic metamaterials like photonic or acoustic crystals through structural engineering^20–33^. PMFs are highly tunable and can reach higher effective field strengths than real magnetic fields.

In 3D, flat bands are challenging to realize, whether via LLs or some other mechanism. For example, stacking 2D quantum Hall systems turns the LLs into 3D bands that are non-flat along the stacking direction, so long as there is nonzero coupling between layers (similar to the original Landau model)^34^. Likewise, if we generalize 2D Dirac particles to Weyl particles in 3D, applying a uniform magnetic field produces chiral LLs that can propagate freely along the field direction^35–38^.

**Fig. 1:**
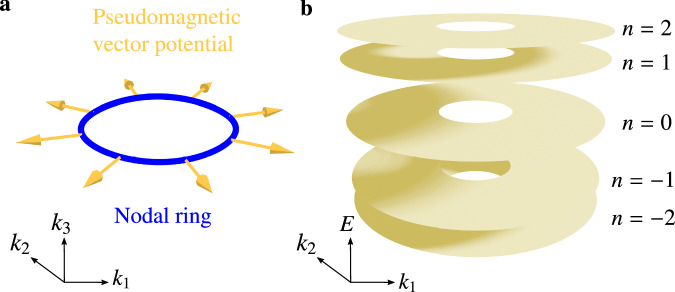
Pseudomagnetic field induced Landau levels in 3D nodal ring systems. **a** Illustration of the pseudomagnetic vector potential (yellow arrows) at different positions of the nodal ring (blue circle). **b** Under the pseudomagnetic vector potential shown in **a**, the nodal ring splits into 3D flat Landau levels.

In this work, we design and experimentally implement a 3D lattice exhibiting LLs that are flat in all three directions. This is accomplished with an acoustic crystal—a synthetic metamaterial through which classical sound waves propagate—that incorporates an inhomogeneous structural distortion. In the absence of the distortion, the band structure contains a circular nodal ring (i.e., a ring in momentum space along which two bands touch)^39,40^. The introduction of the distortion generates a PVP pointing radially from the nodal ring’s center in momentum space, as well as varying in real space (Fig. 1a). The nodal ring spectrum hence splits into 3D flat bands (Fig. 1b), in a manner analogous to the formation of 2D LLs from Dirac points^16^. This 3D flat band spectrum has potential uses for enhancing nonlinearities and accessing interesting 3D phenomena such as inverse Anderson localization^41^. The acoustic crystal design might also help develop solid-state materials hosting 3D LLs, which could access correlated electron phases not found in lower-dimensional flat band systems^42^.

## Results

### Continuum model

In PMF engineering, as performed in strained graphene^16^ and related materials^18,19,24,25^, a lattice distortion shifts a band structure’s nodal points (e.g., Dirac points) in momentum space, which is analogous to applying a vector potential **A**. For instance, a slowly varying (compared to the lattice constant) distortion can be implemented ${{{{{{{\bf{A}}}}}}}}=B{x}_{3}{\hat{{{{{{{{\bf{e}}}}}}}}}}_{1}$ (where *x*_1,2,3_ denotes position coordinates and ${\hat{{{{{{{{\bf{e}}}}}}}}}}_{1,2,3}$ the unit vectors), corresponding to a uniform PMF $\nabla \times {{{{{{{\bf{A}}}}}}}}=B\,{\hat{{{{{{{{\bf{e}}}}}}}}}}_{2}$. A similar manipulation can be applied to nodal lines rather than nodal points^42–44^. Consider the continuum Hamiltonian^45^

1 $$H\left({{{{{{{\bf{k}}}}}}}}\right)=\frac{1}{2{m}_{\rho }}\left({k}_{\rho }^{2}-{k}_{0}^{2}\right){\sigma }_{1}+{v}_{3}{k}_{3}{\sigma }_{2},$$

where **k** = (*k*_1_, *k*_2_, *k*_3_) is 3D momentum vector, *σ*_1,2_ are Pauli matrices, ${k}_{\rho }^{2}={k}_{1}^{2}+{k}_{2}^{2}$, and *m*_*ρ*_, *k*_0_, *v* are positive real parameters. This hosts a nodal ring at *k*_*ρ*_ = *k*_0_, *k*_3_ = 0, with energy *E* = 0^45^. Now, suppose we impose a PVP

2 $${{{{{{{\bf{A}}}}}}}}\left({k}_{1},{k}_{2},{x}_{3}\right)={B}_{0}{x}_{3}{\hat{{{{{{{{\bf{e}}}}}}}}}}_{\rho },$$

where ${\hat{{{{{{{{\bf{e}}}}}}}}}}_{\rho }={k}_{\rho }^{-1}({k}_{1},{k}_{2},0)$ is the radial vector in the plane of the nodal ring. If we treat *x*_3_ as a constant, the Peierls substitution **k** → **k** + **A** shifts the nodal ring’s radius to ${k}_{0}^{{\prime} }={k}_{0}-{B}_{0}{x}_{3}$. For a slow variation, *x*_3_ = −*i*∂/∂*k*_3_, we can expand *H* close to the nodal ring (i.e., $\left\vert {k}_{\rho }-{k}_{0}^{{\prime} }\right\vert \ll {k}_{0}^{{\prime} }$) to obtain

3 $$H\left({{{{{{{\bf{k}}}}}}}}\right) \, \approx \, \frac{{k}_{0}}{{m}_{\rho }}\left({k}_{\rho }+{B}_{0}{x}_{3}-{k}_{0}\right){\sigma }_{1}+{v}_{3}{k}_{3}{\sigma }_{2}.$$

This is a 2D Dirac equation based on coordinates *ρ* and *x*_3_, with a uniform PMF. Its spectrum is ${E}_{n}={{{{{{{\rm{sgn}}}}}}}}(n){\omega }_{c}\,\sqrt{| n| }$, where ${\omega }_{c}=\sqrt{2{v}_{3}{B}_{0}{k}_{0}/{m}_{\rho }}$ and *n* = 0, ± 1, ± 2, … (Supplementary Information section II). Each LL is flat along *k*_*ρ*_, *k*_3_, and the nodal ring plane’s azimuthal coordinate (which *H* does not depend upon).

### Lattice model

**Fig. 2:**
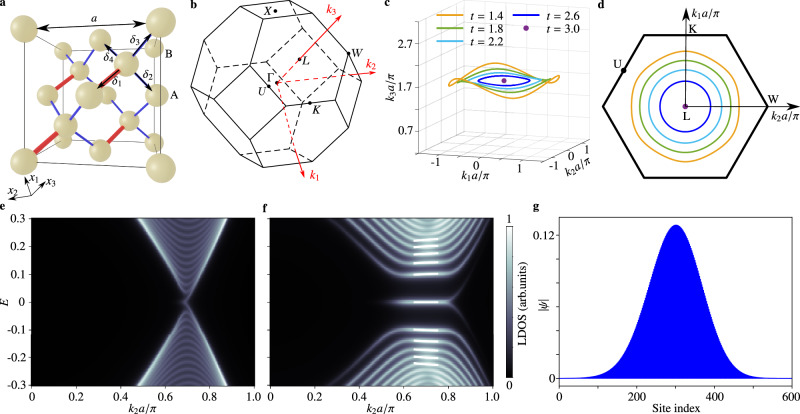
3D Landau levels in an inhomogeneous anisotropic diamond lattice. **a** Cubic cell of the anisotropic diamond lattice. The cubic cell has side length *a*. Red and blue bonds denote nearest-neighbor couplings *t* and $t{\prime}=1$, respectively, and nearest-neighbor sites are separated by the vectors *δ*_*i*_(*i* = 1, 2, 3, 4). The Cartesian coordinate axes are *x*_*i*_(*i* = 1, 2, 3), such that *x*_3_ is parallel to −*δ*_1_, and *x*_2_ is parallel to −*δ*_3_ + *δ*_4_. **b** Schematic of the first Brillouin zone. The reciprocal lattice vectors *k*_*i*_(*i* = 1, 2, 3) are oriented along *LK*, *LW*, and Γ*L*, respectively. **c** Shapes of the nodal ring for various *t*. **d** Projections of the nodal ring onto the *k*_1_−*k*_2_ plane, for the values of *t* used in (**c**). **e** and **f** Local density of states in the *k*_2_ direction for *B* = 0 (**e**) and *B* = 0.0073*a*^−2^ (**f**), calculated using a 600-site chain. Solid white lines in **f** represent the analytically predicted Landau levels. **g** Wavefunction amplitude of the zeroth Landau level at $\left({k}_{1},{k}_{2}\right)=\left(0,0.70\pi /a\right)$.

Following our scheme, the key to realizing 3D LLs is to have a band structure with a circular nodal ring, whose radius can be parametrically varied without losing its circularity. Such a situation arises in a tight-binding model of an anisotropic diamond lattice^46^. As shown in Fig. 2a, the cubic cell has period *a*. There are two sublattices with one *s* orbital per site, and the nearest-neighbor couplings are *t* (red bonds) and ${t}^{{\prime} }$ (blue bonds). The momentum-space lattice Hamiltonian is

4 $$H({{{{{{{\bf{k}}}}}}}})=\left(\begin{array}{cc}0&t{{\rm {e}}}^{i{{{{{{{\bf{k}}}}}}}}\cdot {{{{{{{{\boldsymbol{\delta }}}}}}}}}_{1}}+{t}^{{\prime} }\mathop{\sum }\nolimits_{i=2}^{4}{{\rm {e}}}^{i{{{{{{{\bf{k}}}}}}}}\cdot {{{{{{{{\boldsymbol{\delta }}}}}}}}}_{i}}\\ t{{\rm {e}}}^{-i{{{{{{{\bf{k}}}}}}}}\cdot {{{{{{{{\boldsymbol{\delta }}}}}}}}}_{1}}+{t}^{{\prime} }\mathop{\sum }\nolimits_{i=2}^{4}{{\rm {e}}}^{-i{{{{{{{\bf{k}}}}}}}}\cdot {{{{{{{{\boldsymbol{\delta }}}}}}}}}_{i}}&0\end{array}\right),$$

where ***δ***_1_, …, ***δ***_4_ are the nearest-neighbor displacements shown in Fig. 2a (Supplementary Information section II). The first Brillouin zone is depicted in Fig. 2b. Thereafter, we set ${t}^{{\prime} }=1$ for convenience. This lattice is known to host a nodal ring when 1 < *t* < 3^46^, whereas for *t* > 3 it is a higher-order topological insulator^47,48^. In the former regime, the nodal ring occurs at *E* = 0 and

5 $${K}_{1}^{2}+{K}_{2}^{2}={\left(\frac{2\sqrt{2}}{a}\sqrt{3-t}\right)}^{2},$$

6 $${K}_{3}=\sqrt{3}\frac{\pi }{a},$$

where $\left({K}_{1},{K}_{2},{K}_{3}\right)$ is the position on the nodal ring. The nodal ring forms a circle in momentum space (Supplementary Information Fig. S1, and Fig. 2c, d). Crucially, its radius is determined solely by *t*. We can form any shape of nodal lines in the continuum model^49^, but it is hard to realize circle-shaped nodal rings. In most cases, nodal line semimetals hold either discrete lines^40^ or closed rings with other shapes^40,50–53^.

To generate the 3D LLs, we modulate *t* along the spatial axis *x*_3_ perpendicular to the plane of the ring so that the nodal ring’s radius increases linearly with *x*_3_. This leads to the gauge field (Supplementary Information section II):

7 $${{{{{{{\bf{A}}}}}}}}\left({x}_{3},\phi \right)=B{x}_{3}\left(\begin{array}{c}\cos \phi \\ \sin \phi \\ 0\end{array}\right),$$

with parameter $B=0.4\sqrt{3}\pi /N{a}^{2}$, which controls the magnitude of PVP. Here, *N* is the number of unit cells along the *x*_3_ direction, and *ϕ* is the azimuth angle in the *k*_1_–*k*_2_ plane. Such a PVP splits the original nodal degeneracy into discrete LLs described by ${E}_{n}={{{{{{{\rm{sgn}}}}}}}}(n)\sqrt{\left\vert n\right\vert }{\omega }_{c}$, where ${\omega }_{c}=v\sqrt{2B}$ is the cyclotron frequency (see Fig. 2e, f). While the nonzero LLs are not ideally flat due to the **k**-dependence of the group velocity *v*, the zeroth LL is exactly flat, which indicates a mechanism for generating flat bands in 3D systems. A numerically calculated profile of the zeroth LL is plotted in Fig. 2g, whose localization in bulk is well-captured by the low-energy theory (Supplementary Information Fig. S3).

### Acoustic lattice design

**Fig. 3:**
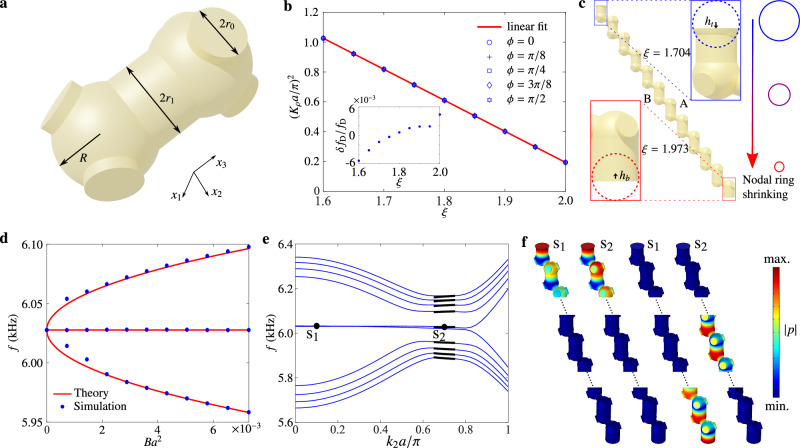
Pseudomagnetic fields and Landau levels in an acoustic nodal-ring crystal. **a** Unit cell of the acoustic lattice, consisting of two sphere cavities connected by cylindrical tubes. The radii of the tubes are *r*_1_ = *ξr*_0_ and *r*_0_, respectively. The radius of the sphere cavity is *R*. **b** Plot of the square of the nodal ring’s radius ${K}_{\rho }=\sqrt{{K}_{1}^{2}+{K}_{2}^{2}}$ against the geometry parameter *ξ* for different polar angles *ϕ*. Blue markers and the red line represent the data and the linear fit, respectively. The lower inset displays the nodal ring’s frequency variation when *ξ* changes. **c** Schematic of a 12-layer inhomogeneous acoustic lattice. The value of *ξ* gradually changes along the *x*_3_ direction, which leads to the ring shrinking and induces a pseudomagnetic field. To realize flat drumhead surface states, the top and bottom resonators are cut by *h*_t_ and *h*_b_, respectively. **d** Eigenfrequencies of the first three Landau levels at $\left({k}_{1},{k}_{2}\right)=\left(0,0.70\pi /a\right)$ for acoustic lattices under different pseudomagnetic fields. The simulation results (blue dots) are well predicted by the theoretical model (red curves). **e** Dispersion along *k*_2_ for an inhomogeneous acoustic lattice with 300 layers and *B* = 0.0073*a*^−2^. Black lines represent analytically predicted Landau levels. **f** Pressure amplitude distributions for the four eigenmodes labeled in (**e**). Both s_1_ and s_2_ are two-fold degenerate. One eigenmode localizes at the top surface, whereas the other moves from bottom to bulk as *k*_2_ increases. The plots only display the chain’s top, middle, and bottom parts and omit other regions where sound pressure is neglectable.

Next, we design an acoustic diamond lattice to realize the above phenomenon. Figure 3a shows the acoustic unit cell, which consists of two sphere cavities connected by cylindrical tubes with radii *r*_1_ or *r*_0_. The whole structure is filled with air and covered by hard walls. Here, the two-sphere cavities act as two sublattices (denoted as “A" and “B"; see Fig. 3c), and the cylindrical tubes control the coupling strengths. We define a dimensionless parameter *ξ* = *r*_1_/*r*_0_ which describes the anisotropic strength of couplings. Like the tight-binding model, this acoustic lattice hosts a circular-like nodal ring in its band structure. We numerically find that the square of the radius of the nodal ring is controlled by a single parameter *ξ* and scales linearly with *ξ* (Fig. 3b). Moreover, for different values of *ξ*, the frequency of the nodal ring remains almost unchanged (the inset to Fig. 3b). We can straightforwardly engineer PMFs in this acoustic lattice with these nice properties. The supercell of the designed structure is schematically illustrated in Fig. 3c, where *ξ* gradually varies along the *x*_3_ direction. This variation makes the radius of the nodal ring linearly dependent on space coordinates, similar to the case in the tight-binding model. We cut part of the top and bottom cavities to tune on-site potential. After precisely controlling *h*_t_ and *h*_b_, the drumhead surface state is almost flat (Fig. 3e), which is an advantage in the drumhead state measurement.

We numerically test our design using a lattice with 300 layers along the *u*_3_ direction. Figure 3d plots the frequencies of *n* = {−1, 0, 1} LLs under different PMFs. As can be seen, the spacings between the LLs follow well with the theoretical curve ${E}_{n}={{{{{{{\rm{sgn}}}}}}}}(n)\sqrt{\left\vert n\right\vert }{\omega }_{c}$. Figure 3e shows the dispersion along the *k*_2_ direction under *B* = 0.0073*a*^−2^, where the acoustic LLs are clearly presented. Notably, the zeroth LL is exponentially localized in bulk and is smoothly connected to the surface modes (Fig. 3f). All these numerical results are consistent with the low-energy theory and tight-binding calculations.

### Experiment

**Fig. 4:**
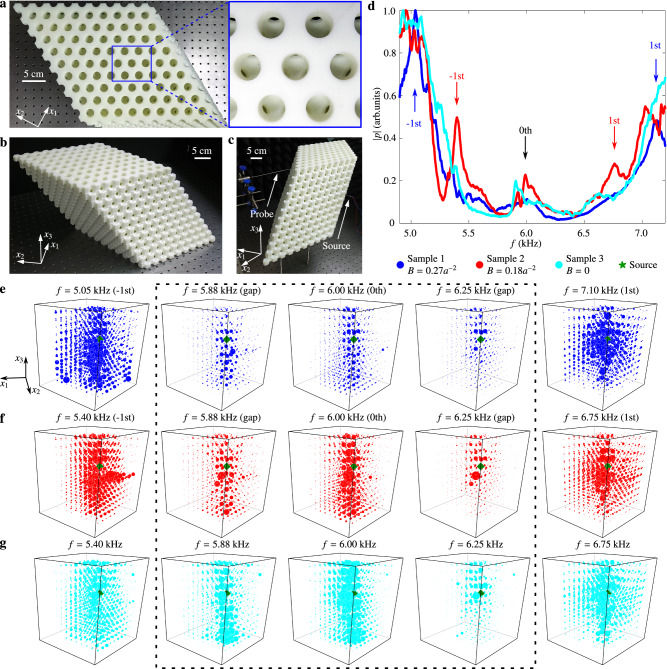
Experimental detection of the acoustic Landau levels. **a** The top view of the sample, with 11 × 11 sites on *x*_1_*x*_2_ plane and 12 layers along *x*_3_ direction, which induce pseudomagnetic fields under *B* = 0.27*a*^−2^, *B* = 0.18*a*^−2^, *B* = 0 for samples 1–3, respectively. **b** A photo of the sample. Sphere cavities are connected by tubes. **c** The photo of the experiment setup. The probe is ensembled on the mechanical arm and controlled by a stepping motor. **d** Measured acoustic pressure spectra at the same bulk site for samples 1 (blue), 2 (red), and 3(cyan). The black arrow indicates frequency *n* = 0 Landau levels for samples 1 and 2. The blue (red) arrows indicate frequencies of *n* = {−1, 1} Landau levels for samples 1(2). **e** Measured acoustic pressure distributions for the *n* = {−1, 0, 1} Landau levels and two gap frequencies in sample 1. The radii of the blue spheres are proportional to the acoustic pressure. **f** Measured acoustic pressure distributions for the *n* = {−1, 0, 1} Landau levels and two gap frequencies in sample 2. The radii of the red spheres are proportional to the acoustic pressure. **g** Measured acoustic pressure distributions with five frequencies in sample 3. The radii of the cyan spheres are proportional to the acoustic pressure. The green marker denotes the sound source’s position, located at the sample’s center.

To observe the LLs experimentally, we fabricate two 12-layer samples with stronger PMFs under *B* = 0.27*a*^−2^, *B* = 0.18*a*^−2^, and one sample with *B* = 0 for comparison, as shown in Fig. 4a–c. Figure 4a shows the top layer of the sample, and Fig. 4c shows the experiment setup. The stepping motor controls the mechanical arm, allowing precise field mapping. The strong PMF leads to LLs with wide-space spacing to be measured in a simple pump-and-probe experiment (see the “Methods” subsection “Experimental measurements”). Figure 4d plots the measured spectrum when the source and the probe are placed at two bulk sites (Supplementary Information Fig. S8). As can be seen, there are pronounced peaks at the corresponding frequencies of the predicted LLs.

To visualize the effect of the PMF, we plot the acoustic field distributions at several representative frequencies corresponding to the frequencies of the *n* = {−1, 0, 1} LLs or the middle frequencies between these LLs. As shown in Fig. 4e, f, the excited fields spread a noticeable area when the source operates at LL frequencies. In contrast, the excited fields are highly confined to the source position at midgap frequencies.

Furthermore, we compare the three samples’ acoustic field distributions at Dirac frequency and gap frequencies. As shown in the 2nd to the 4th columns in Fig. 4e–g, the stronger the PMF, the more localized the field. Such a sharp comparison is a direct consequence of the Landau quantization of the acoustic bands.

**Fig. 5:**
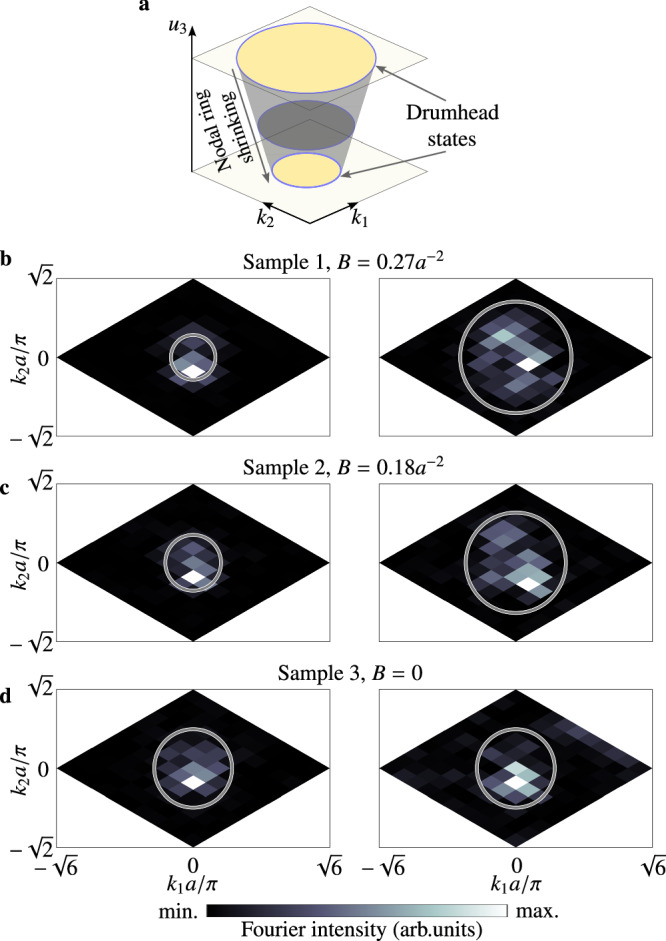
Pseudomagnetic field modified drumhead surface states. **a** Illustration of the drumhead states at the top and bottom surface. **b**–**d** Measured Fourier intensity at the bottom (top) surface at 6.13 kHz for three samples. The gray circles denote the projections of the nodal ring near the surfaces.

Conventional nodal-line crystals are accompanied by the drumhead surface states, which are bounded by the projection of the bulk nodal line^39,40,53^. In the presence of the PMF, we find that the drumhead surface states are also modified. As illustrated in Fig. 5a, due to the spatial variation of the nodal ring’s radius, the momentum space area of the drumhead surface states at the top and bottom surfaces are different (Supplementary Information Fig. S4). To see this effect, we measure the acoustic fields at the top and bottom surfaces for three samples with different PMF strengths, and the corresponding Fourier spectra are given in Fig. 5b–d. Due to the small dispersion of drumhead surface states, the drumhead surface state frequency is at 6.13 kHz, shifting slightly from the zeroth LL. As shown in Fig. 5b, c, the surface states at the top surface indeed occupy a larger area in the momentum space compared to those at the bottom.

## Discussion

To sum up, we have theoretically proposed and experimentally demonstrated the generation of PMFs in 3D nodal ring systems. Our results open a route to studying the physics of artificial gauge fields in gapless systems beyond Dirac and Weyl semimetals. From the perspective of wave manipulation, the PMF-induced LLs provide a method to generate flat bands in 3D systems^10^, which could be helpful in sound trapping, energy harvest, and slow-wave devices. In future studies, it would be interesting to investigate the effects of other forms of PMF beyond the constant one^31^ and the interactions between PMFs and different types of band degeneracies, such as nodal link, nodal knot, and nodal surfaces. Extending the idea to photonic and electronic systems is also highly desired, where nonlinear and correlated physics can be studied.

## Methods

### Tight-binding calculations

The nodal ring structures in Fig. 2c, d are obtained by directly diagonalizing the lattice Bloch Hamiltonian (i.e., Eq. (4)) and then tracing the degenerate eigenvalues. The local density of states of plots (Fig. 2e, f) is calculated via Green’s function:

8 $$G=\frac{1}{E+i\gamma -H}$$

as -$\,{{\mbox{Im}}}\,\left({\sum }_{i\in 400{{{{{{{\rm{bulk}}}}}}}}{{{{{{{\rm{sites}}}}}}}}}{G}_{ii}\right)/\pi$. Here *H* is the Hamiltonian for a supercell with 600 sites along the *x*_3_ direction and is periodic in the other two directions and *γ* = 0.01. In Fig. 2e, we fix *t* unchanged, and the radius of the nodal ring is 0.70*π*/*a*. In Fig. 2f, The coupling parameter *t* varies along *x*_3_, and the radius of the nodal ring expands from 0.5*π*/*a* (bottom) to 0.9*π*/*a* (top).

### Acoustic lattice design

The side length of the cubic cell is set to be *a* = 4 cm, while other structural parameters are all tunable. All tunable structural parameters are controlled by the dimensionless parameter *ξ*. Specifically, the radii of the tubes are given by *r*_1_ = *ξr*_0_ and *r*_0_ with *r*_0_ = 0.4 cm, and the sphere’s radius is *R* = 0.8 cm.

### Numerical simulations

All simulations are performed using the acoustic module of COMSOL Multiphysics, which is based on the finite-element method. The photosensitive resin used for sample fabrication is set as the hard boundary due to its large impedance mismatch with air. The real sound speed at room temperature is *c*_0_ = 346 m/s. The density of air is set to be 1.8 kg/m^3^. The results in Fig. 3b are obtained by computing the band structure of one unit cell, with the Floquet boundary condition applied to all directions. The data points are selected by scanning the dispersion along *k*_1_, *k*_2_, *k*_3_ and tracing the degenerate points. We note this is a good measure of the nodal ring’s radius due to its circular shape (Fig. 3b and Supplementary Information Fig. S5). In the simulations of Fig. 3d–f, a supercell with 300 layers along the *x*_3_ direction is used. In Fig. 3e, f, *ξ* changes from 1.973 to 1.704 from bottom to top. Accordingly, the radius of the nodal ring expands from 0.5*π*/*a* to 0.9*π*/*a*. To realize flat drumhead surface states, the top and bottom resonators are cut by *h*_t_ = 0.246*r*_0_ and *h*_b_ = 0.216*r*_0_, respectively. More simulations can be found in Supplemental Information.

### Sample details

The sample is fabricated via 3D printing technology with a fabrication error of 0.1 mm. The triclinic sample has a side length of 28.28 cm × 28.28 cm × 32.01 cm and contains 2904 sphere cavities and several waveguides. All spheres at the surface are cut in half to mimic radiation boundary conditions. During the measurement, the sample is surrounded by sound-absorbing sponges to reduce the reflection from the sample boundaries. When we measure the top (bottom) drumhead surface states, the top (bottom) surface is covered by an acrylic plate (Supplementary Information Fig. S10). All three samples have 12 layers. The parameters of the three samples are listed in Table 1.

**Table 1:** Parameters of three samples^a^

Sample	*ξ*	*k*_*ρ*_*a*/*π*	*h*_t_	*h*_b_
1	1.612–2.016	1.0–0.4	0.258*r*_0_	0.148*r*_0_
2	1.704–1.973	0.9–0.5	0.246*r*_0_	0.216*r*_0_
3	1.858	0.7	0.278*r*_0_	0.226*r*_0_

^a^The meaning of *ξ*, *h*_t_, and *h*_b_ defines the geometry of the unit cell, and they are illustrated in Fig. 3. *k*_*ρ*_ describes the radius of the nodal ring.

### Experimental measurements

In the LL experiment, a broadband sound signal (4–8 kHz) is launched from a narrow long tube in Fig. 4c (diameter of about 0.3 cm and length of 35 cm) that is inserted into the centermost site, which acts as a point-like sound source for the wavelength focused here. The pressure of each site is detected by a microphone (Brüel&Kjær Type 4961) adhered to a long tube (diameter of about 0.2 cm and length of 35 cm). The signal is recorded and frequency-resolved by a multi-analyzer system (Brüel&Kjær 3160-A-022 module). In the drumhead state experiment, the source is located at the 2nd (12th) layer when we measure the bottom (top) drumhead state (Supplementary Information Fig. S10). Other sets are the same as before.

### Data analysis

In the LL experiment, pressure data of the outermost sites (cut sites and sites that connect them directly) are discarded. The source’s frequency spectrum normalizes all pressure data before they are used to create the plots in Fig. 4. In the drumhead state experiment, all data are processed by Fourier transformation.

### Supplementary information

Supplementary Information

Peer review file

## Data Availability

The experimental data are available in the data repository for Nanyang Technological University at this link (10.21979/N9/RS60NB). Other data supporting this study’s findings are available from the corresponding authors upon reasonable request.
