# Supplementary material for: Three-dimensional flat Landau levels in an inhomogeneous acoustic crystal
Source: Nat Commun. 2024 Mar 11;15:2174. doi: 10.1038/s41467-024-46517-z (PMC10928213; doi:10.1038/s41467-024-46517-z)
Supplement: Supplementary file 1 — Supplementary Information [file 41467_2024_46517_MOESM1_ESM.pdf]

Supplementary Information for
"Three-dimensional flat Landau levels in an inhomogeneous acoustic crystal"

Zheyu Cheng,[1,*] Yi-jun Guan,[2,3,*] Haoran Xue,[4,†] Yong Ge,[2] Ding Jia,[2] Yang Long,[1]
Shou-qi Yuan,[2] Hong-xiang Sun,[2,3,‡] Yidong Chong,[1,5,§] and Baile Zhang[1,5,¶]

[1]*Division of Physics and Applied Physics,*
School of Physical and Mathematical Sciences,
Nanyang Technological University, Singapore 637371, Singapore

[2]*Research Center of Fluid Machinery Engineering and Technology,*
School of Physics and Electronic Engineering,
Jiangsu University, Zhenjiang 212013, China

[3]*State Key Laboratory of Acoustics, Institute of Acoustics,*
Chinese Academy of Sciences, Beijing 100190, China

[4]*Department of Physics, The Chinese University of Hong Kong, Shatin, Hong Kong SAR, China*

[5]*Centre for Disruptive Photonic Technologies,*
Nanyang Technological University, Singapore, 637371, Singapore

* These authors contributed equally to this work

† haoranxue@cuhk.edu.hk

‡ jsdxshx@ujs.edu.cn

§ yidong@ntu.edu.sg

¶ blzhang@ntu.edu.sg

I. CONTINUUM MODEL

This section provides detailed derivations of Landau levels (LLs) in the continuum model.

After the Peierls substitution $\mathbf{k} \to \mathbf{k} + \mathbf{A}$, the continuum Hamiltonian is

$$H(\mathbf{k}, \mathbf{A}) = \frac{1}{2m_\rho}\left[\left(k_\rho + B_0 x_3\right)^2 - k_0^2\right]\sigma_1 + v_3 k_3 \sigma_2. \tag{S1-1}$$

The equation determines the nodal ring

$$K_\rho = k_0 - B_0 x_3, \quad K_3 = 0. \tag{S1-2}$$

For a slow variation, $x_3 = -i\partial/\partial k_3$, we can expand H close to the nodal ring (i.e., $\left|k_\rho - K_\rho\right| \ll K_\rho$),

$$\begin{aligned} H(\mathbf{k}, \mathbf{A}) =& \frac{1}{2m_\rho}\left(k_\rho + B_0 x_3 + k_0\right)\left(k_\rho + B_0 x_3 - k_0\right)\sigma_1 + v_3 k_3 \sigma_2 \\ \approx& \frac{k_0}{m_\rho}\left(k_\rho + B_0 x_3 - k_0\right)\sigma_1 + v_3 k_3 \sigma_2. \end{aligned} \tag{S1-3}$$

For later convenience, we denote

$$h_1 = \frac{k_0}{m_\rho}\left(k_\rho + B_0 x_3 - k_0\right), \quad h_2 = v_3 k_3. \tag{S1-4}$$

The canonical quantization in the polar coordinate is

$$k_\rho = -i\left(\partial_\rho + \frac{1}{2\rho}\right), \quad k_\phi = -i\partial_\phi, \quad k_3 = -i\partial_3. \tag{S1-5}$$

From Eqs. (S1-4)(S1-5), Eq. (S1-3) can be written as

$$H = \omega_c \begin{pmatrix} 0 & a^\dagger \\ a & 0 \end{pmatrix}. \tag{S1-6}$$

Here we define the canonically conjugated ladder operators $a = \frac{1}{\omega_c}(h_1 + ih_2)$ and $a^\dagger = \frac{1}{\omega_c}(h_1 - ih_2)$, which satisfy

$$1 = \left[a, a^\dagger\right] = \frac{2i}{\omega_c^2}\left[h_2, h_1\right]. \tag{S1-7}$$

From Eqs. (S1-4)(S1-5)(S1-7), we have

$$\omega_c = \sqrt{2v_3 \frac{k_0}{m_\rho} B_0}. \tag{S1-8}$$

The solution of Eq. (S1-6) is

$$H\begin{pmatrix} |n\rangle \\ \pm|n-1\rangle \end{pmatrix} = \pm\sqrt{n}\begin{pmatrix} |n\rangle \\ \pm|n-1\rangle \end{pmatrix}, \quad H\begin{pmatrix} |n\rangle \\ 0 \end{pmatrix} = 0. \tag{S1-9}$$

Then, the LLs are determined by

$$E_n = \mathrm{sgn}(n)\,\omega_c\sqrt{|n|}, \tag{S1-10}$$

which are flat for the zeroth LL and dispersion-free along the x_3 direction.

II. TIGHT-BINDING MODEL

In this section, we provide detailed derivations and calculations of the shape of the nodal ring, the effective Hamiltonian, and LLs in the anisotropic diamond lattice model.

A. Shape of the nodal ring

The Bloch Hamiltonian of the anisotropic diamond lattice can be written as

$$H(\mathbf{k}) = \begin{pmatrix} 0 & te^{i\mathbf{k}\cdot\boldsymbol{\delta}_1} + \sum_{i=2}^{4} e^{i\mathbf{k}\cdot\boldsymbol{\delta}_i} \\ te^{-i\mathbf{k}\cdot\boldsymbol{\delta}_1} + \sum_{i=2}^{4} e^{-i\mathbf{k}\cdot\boldsymbol{\delta}_i} & 0 \end{pmatrix}, \tag{S2-1}$$

where $\boldsymbol{\delta}_1 = \left(0, 0, -\frac{\sqrt{3}}{4}\right)a$, $\boldsymbol{\delta}_2 = \left(\frac{\sqrt{6}}{6}, 0, \frac{\sqrt{3}}{12}\right)a$, $\boldsymbol{\delta}_3 = \left(-\frac{\sqrt{6}}{12}, -\frac{\sqrt{2}}{4}, \frac{\sqrt{3}}{12}\right)a$, $\boldsymbol{\delta}_4 = \left(-\frac{\sqrt{6}}{12}, \frac{\sqrt{2}}{4}, \frac{\sqrt{3}}{12}\right)a$, as shown in Fig. 1 in the main text. The nodal points (K_1, K_2, K_3) are determined by $te^{i\mathbf{k}\cdot\boldsymbol{\delta}_1} + \sum_{i=2}^{4} e^{i\mathbf{k}\cdot\boldsymbol{\delta}_i} = 0$, which leads to the following equations:

$$\begin{aligned} t + \cos\left(\frac{K_1 + \sqrt{2}K_3}{\sqrt{6}}a\right) + \cos\left(\frac{-K_1 - \sqrt{3}K_2 + 2\sqrt{2}K_3}{2\sqrt{6}}a\right) + \cos\left(\frac{-K_1 + \sqrt{3}K_2 + 2\sqrt{2}K_3}{2\sqrt{6}}a\right) &= 0, \\ \sin\left(\frac{K_1 + \sqrt{2}K_3}{\sqrt{6}}a\right) + \sin\left(\frac{-K_1 - \sqrt{3}K_2 + 2\sqrt{2}K_3}{2\sqrt{6}}a\right) + \sin\left(\frac{-K_1 + \sqrt{3}K_2 + 2\sqrt{2}K_3}{2\sqrt{6}}a\right) &= 0. \end{aligned} \tag{S2-2}$$

Expand Eq. (S2-2) to second order near the L point $\left(0, 0, \sqrt{3}\frac{\pi}{a}\right)$, we can have the following approximate solution:

$$K_1^2 + K_2^2 = \frac{8}{a^2}(3 - t), \tag{S2-3}$$

$$K_3 = \sqrt{3}\frac{\pi}{a}, \tag{S2-4}$$

which describe a circle in the k_1–k_2 plane. Figure S1 displays the comparisons between the numerically obtained nodal ring and a circle whose radius is $\sqrt{8(3-t)}/a$. As can be seen, the shape of the nodal ring can be well approximated as a circle. When t is close to 3 (i.e., the radius of the nodal ring is small), the nodal ring is well described by the solution in Eqs. (S2-3)–(S2-4). As t decreases, the discrepancy between the real nodal ring shape and the approximate solution gradually increases due to increasing the nodal ring's radius. However, even when t is relatively small, the nodal ring remains circular in the k_1–k_2 plane, which is the key to constructing LLs with equal spacing for all nodal points. It is also worth noting that the fluctuation of the nodal ring in the k_3 direction will not be a problem in engineering the LLs. This is because such a fluctuation,

which corresponds to the third component of the gauge field in a lattice with varying gauge fields along the third dimension, will not contribute to the pseudomagnetic field (PMF).

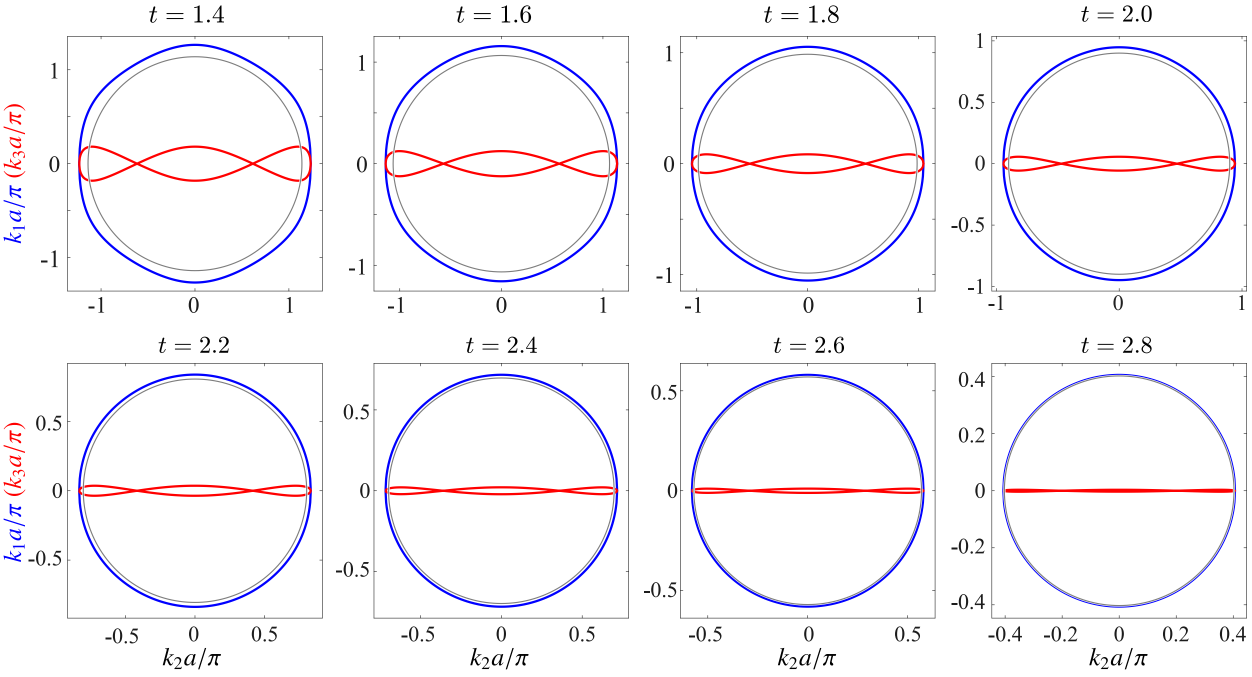

FIG. S1. Plot of the relationship between k_1, k_2 and k_3. Comparison between $k_1 - k_2$ projected nodal ring (blue curve) and $k_1^2 + k_2^2 = \frac{8}{a^2}(3 - t)$ (gray circle). Red curves shows the $k_2 - k_3$ plane projected nodal ring, which proves that k_3 component is much less than k_1 and k_2 components.

B. Effective Hamiltonian

To get the effective Hamiltonian of the nodal ring, we expand Eq. (S2-1) to linear order near the degenerate point (K_1, K_2, K_3). Denote $(k_1, k_2, k_3) = (K_1, K_2, K_3) + (\kappa_1, \kappa_2, \kappa_3)$. Then, the off-diagonal element of the Hamiltonian can be written as

$$
\begin{aligned}
H_{12}(\boldsymbol{\kappa}) \approx{}& t e^{-i\frac{\sqrt{3}}{4}K_3 a}\left(1 - i\frac{\sqrt{3}}{4}\kappa_3 a\right) + e^{i\frac{1}{12}(2\sqrt{6}K_1 + \sqrt{3}K_3)a}\left[1 + i\frac{1}{12}\left(2\sqrt{6}\kappa_1 + \sqrt{3}\kappa_3\right)a\right] \\
&+ e^{i\frac{1}{12}(-\sqrt{6}K_1 - 3\sqrt{2}K_2 + \sqrt{3}K_3)a}\left[1 + i\frac{1}{12}\left(-\sqrt{6}\kappa_1 - 3\sqrt{2}\kappa_2 + \sqrt{3}\kappa_3\right)a\right] \\
&+ e^{i\frac{1}{12}(-\sqrt{6}K_1 + 3\sqrt{2}K_2 + \sqrt{3}K_3)a}\left[1 + i\frac{1}{12}\left(-\sqrt{6}\kappa_1 + 3\sqrt{2}\kappa_2 + \sqrt{3}\kappa_3\right)a\right] \\
={}& -\frac{1}{\sqrt{6}}\left[\sin\left(\frac{2\sqrt{6}K_1 + \sqrt{3}K_3}{12}a\right) + \sin\left(\frac{\sqrt{6}K_1 - \sqrt{3}K_3}{12}a\right)\cos\left(\frac{K_2}{2\sqrt{2}}a\right)\right]\kappa_1 a - \frac{1}{\sqrt{2}}\left[\cos\left(\frac{\sqrt{6}K_1 - \sqrt{3}K_3}{12}a\right)\sin\left(\frac{K_2}{2\sqrt{2}}a\right)\right]\kappa_2 a \\
&+ \frac{1}{\sqrt{3}}\left[-\sin\left(\frac{2\sqrt{6}K_1 + \sqrt{3}K_3}{12}a\right) + 2\sin\left(\frac{\sqrt{6}K_1 - \sqrt{3}K_3}{12}a\right)\cos\left(\frac{K_2}{2\sqrt{2}}a\right)\right]\kappa_3 a \\
&- \frac{i}{\sqrt{6}}\left[-\cos\left(\frac{2\sqrt{6}K_1 + \sqrt{3}K_3}{12}a\right) + \cos\left(\frac{\sqrt{6}K_1 - \sqrt{3}K_3}{12}a\right)\cos\left(\frac{K_2}{2\sqrt{2}}a\right)\right]\kappa_1 a \\
&+ \frac{i}{\sqrt{2}}\left[\sin\left(\frac{\sqrt{6}K_1 - \sqrt{3}K_3}{12}a\right)\sin\left(\frac{K_2}{2\sqrt{2}}a\right)\right]\kappa_2 a + \frac{i}{\sqrt{3}}\left[\cos\left(\frac{2\sqrt{6}K_1 + \sqrt{3}K_3}{12}a\right) + 2\cos\left(\frac{\sqrt{6}K_1 - \sqrt{3}K_3}{12}a\right)\cos\left(\frac{K_2}{2\sqrt{2}}a\right)\right]\kappa_3 a. \qquad \text{(S2-5)}
\end{aligned}
$$

The effective Hamiltonian can then be written in a compact form:

$$H_{\text{eff}}(\boldsymbol{\kappa}) = \sum_{i,j} \sigma_i v_{ij} \kappa_j \qquad (i = 1,2,\ j = 1,2,3), \tag{S2-6}$$

where $\sigma_i (i = 1, 2)$ are Pauli matrices and

$$\boldsymbol{v}^{\mathrm{T}} = \begin{pmatrix} -\frac{1}{\sqrt{6}}\left[\sin\left(\frac{2\sqrt{6}K_1+\sqrt{3}K_3}{12}a\right) + \sin\left(\frac{\sqrt{6}K_1-\sqrt{3}K_3}{12}a\right)\cos\left(\frac{K_2}{2\sqrt{2}}a\right)\right]a & \frac{1}{\sqrt{6}}\left[-\cos\left(\frac{2\sqrt{6}K_1+\sqrt{3}K_3}{12}a\right) + \cos\left(\frac{\sqrt{6}K_1-\sqrt{3}K_3}{12}a\right)\cos\left(\frac{K_2}{2\sqrt{2}}a\right)\right]a \\ -\frac{1}{\sqrt{2}}\left[\cos\left(\frac{\sqrt{6}K_1-\sqrt{3}K_3}{12}a\right)\sin\left(\frac{K_2}{2\sqrt{2}}a\right)\right]a & -\frac{1}{\sqrt{2}}\left[\sin\left(\frac{\sqrt{6}K_1-\sqrt{3}K_3}{12}a\right)\sin\left(\frac{K_2}{2\sqrt{2}}a\right)\right]a \\ \frac{1}{\sqrt{3}}\left[-\sin\left(\frac{2\sqrt{6}K_1+\sqrt{3}K_3}{12}a\right) + 2\sin\left(\frac{\sqrt{6}K_1-\sqrt{3}K_3}{12}a\right)\cos\left(\frac{K_2}{2\sqrt{2}}a\right)\right]a & -\frac{1}{\sqrt{3}}\left[\cos\left(\frac{2\sqrt{6}K_1+\sqrt{3}K_3}{12}a\right) + 2\cos\left(\frac{\sqrt{6}K_1-\sqrt{3}K_3}{12}a\right)\cos\left(\frac{K_2}{2\sqrt{2}}a\right)\right]a \end{pmatrix}. \tag{S2-7}$$

The effective Hamiltonian only contains two of the three Pauli matrices, which is expected for a nodal line system. Figure S2 shows the calculated dispersions using the effective Hamiltonian (red dots) and the lattice Hamiltonian (blue lines), which match well with each other.

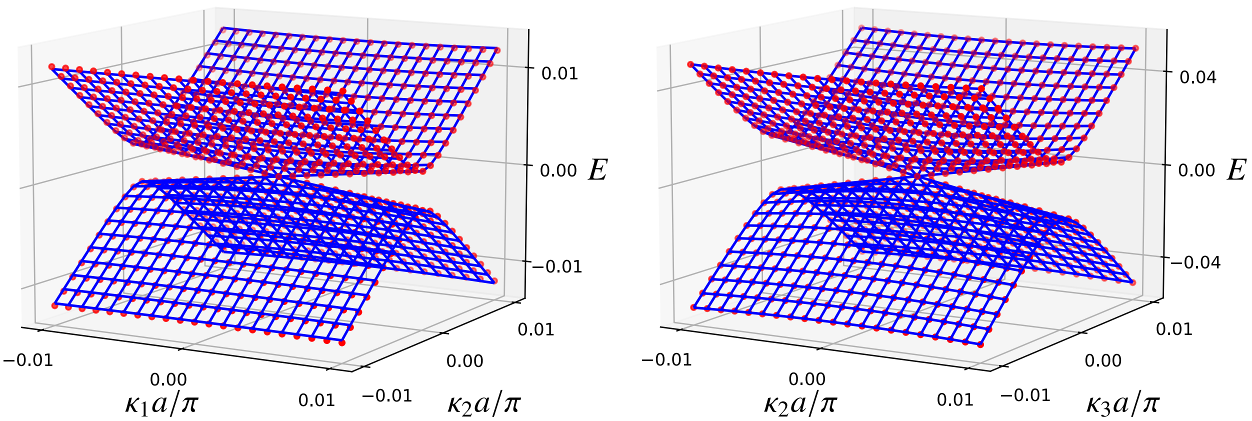

FIG. S2. Plot of projected band structure in $\kappa_1 - \kappa_2$ plane (left) and $\kappa_2 - \kappa_3$ plane (right) near degenerate point $(0, \pi/2, 0)$ with $t = 1 + 2\cos\left(\frac{\pi}{4\sqrt{2}}\right)$. The blue lines and red dots are calculated from Hamiltonian (Eq. S2-1) and effective Hamiltonian (Eq. S2-6), respectively.

C. Pseudomagnetic field and Landau levels

This subsection provides detailed derivations and more numerical results for the PMF-induced LLs.

Consider an inhomogeneous lattice that is periodic in x_1 and x_2 directions and has spatially varying t in x_3 direction. The lattice consists of N unit cells along the x_3 direction. As t changes in space, the nodal ring shrinks/expands accordingly. Such a movement of the nodal points can be regarded as a pseudomagnetic vector potential (PVP) $\mathbf{A}(x_3, \phi) = \delta\mathbf{K}$. Here ϕ is the azimuth angle in

the k_1–k_2 plane. $\delta\mathbf{K}$ is the momentum shift, a function of both j and ϕ. We denote $K_\rho = \sqrt{K_1^2 + K_2^2}$ as the radius of the nodal ring (Note we have shown in the first subsection that the shape of a nodal ring in k_1–k_2 plane is almost identical to a circle). To implement a uniform PMF in the real space, we require K_ρ to change along x_3 direction according to the following relation:

$$K_\rho = \frac{1}{a}\left(\alpha_1 \frac{\sqrt{3}x_3}{Na} + \alpha_2\right). \tag{S2-8}$$

Here α_1 and α_2 are constants that can be used to tune the strength of the PMF. In the main text, we choose $\alpha_1 = 0.4\pi$, $\alpha_2 = 0.5\pi$. In the Supplementary Information, we choose $\alpha_1 = \pi$, $\alpha_2 = 0$ for more flat LLs. In numerical calculations, we first numerically retrieve the relationship between K_ρ and t and then adjust t along x_3 direction accordingly to implement Eq. (S2-8). Under such a nodal ring's radius modulation, the first two components of the PVP are

$$\begin{aligned} A_1(j,\phi) &= \alpha_1 \frac{\sqrt{3}x_3}{Na^2} \cos\phi, \\ A_2(j,\phi) &= \alpha_1 \frac{\sqrt{3}x_3}{Na^2} \sin\phi. \end{aligned} \tag{S2-9}$$

From Eq. (S2-9), the parameter

$$B = \frac{\sqrt{3}\alpha_1}{Na^2}. \tag{S2-10}$$

Next, we show the consequences of the PMF. After we implement the PVP $\mathbf{A}$, the effective Hamiltonian Eq. (S2-6) becomes

$$H_{\text{eff}} = h_1\sigma_1 + h_2\sigma_2, \tag{S2-11}$$

$$h_1 = v_{11}(\kappa_1 + A_1) + v_{12}(\kappa_2 + A_2) + v_{13}(-i\partial_3 + A_3), \tag{S2-12}$$

$$h_2 = v_{21}(\kappa_1 + A_1) + v_{22}(\kappa_2 + A_2) + v_{23}(-i\partial_3 + A_3). \tag{S2-13}$$

We replace κ_3 with $-i\partial_3$ since the translation symmetry along x_3 direction is spoiled due to the slowly varying t. The Eigen equation can then be written as

$$H_{\text{eff}}\begin{pmatrix}\psi_B\\ \psi_A\end{pmatrix} = \omega_c \begin{pmatrix}0 & a^\dagger\\ a & 0\end{pmatrix}\begin{pmatrix}\psi_B\\ \psi_A\end{pmatrix} = E\begin{pmatrix}\psi_B\\ \psi_A\end{pmatrix}. \tag{S2-14}$$

Here we define the canonically conjugated ladder operators $a = \frac{1}{\omega_c}(h_1 + ih_2)$ and $a^\dagger = \frac{1}{\omega_c}(h_1 - ih_2)$, which satisfy

$$1 = \left[a, a^\dagger\right] = \frac{2i}{\omega_c^2}[h_2, h_1]. \tag{S2-15}$$

The parameter ω_c is the analogous cyclotron frequency given by

$$\omega_c^2 = \frac{\alpha_1}{N}\left\{\sqrt{6}\cos\phi\sin\left(\frac{\sqrt{6}}{4}K_1 a\right)\cos\left(\frac{\sqrt{2}}{4}K_2 a\right) + \sqrt{2}\sin\phi\left[\cos\left(\frac{\sqrt{6}}{4}K_1 a\right)\sin\left(\frac{\sqrt{2}}{4}K_2 a\right) + \sin\left(\frac{\sqrt{2}}{2}K_2 a\right)\right]\right\}. \tag{S2-16}$$

Separating Eq. (S2-14), we have:

$$\omega_c^2 a^\dagger a \psi_B = E^2 \psi_B, \tag{S2-17}$$

$$\omega_c^2 a a^\dagger \psi_A = E^2 \psi_A. \tag{S2-18}$$

As $a^\dagger a$ is the number operator, the LLs are determined by

$$E_n = \operatorname{sgn}(n)\,\omega_c\sqrt{|n|}. \tag{S2-19}$$

Although ω_c is a function of ϕ, its dependence on ϕ is relatively weak. Thus, the spacings between the LLs are uniform along the ϕ direction, which is evident in the numerical calculations in Fig. S3.

Then, we calculate the wavefunction. We focus on the zeroth LL at $(0, k_2, 0)$ with $k_2 > 0$. Near the nodal point $\left(0, K_\rho, 0\right)$, we have

$$h_1 = \frac{1}{\sqrt{2}}\sin\frac{K_\rho a}{2\sqrt{2}}\left(\kappa_2 + A_2\right)a, \tag{S2-20}$$

$$h_2 = -i\frac{1}{\sqrt{3}}\left(1 + 2\cos\frac{K_\rho a}{2\sqrt{2}}\right)\partial_3 a. \tag{S2-21}$$

Substituting Eqs. (S2-20)–(S2-21) into Eqs. (S2-17)–(S2-18), we have

$$\frac{1}{\omega_c}\left(h_1 + i h_2\right)\psi_{B0} = 0, \tag{S2-22}$$

$$\psi_{A0} = 0. \tag{S2-23}$$

By using $x_3 = \frac{ja}{\sqrt{3}}$, further simplification leads to

$$\partial_j \psi_{B0} = -\sqrt{\frac{1}{2}}\frac{\sin\frac{K_\rho a}{2\sqrt{2}}}{1 + 2\cos\frac{K_\rho a}{2\sqrt{2}}}\left(\kappa_2 a + \frac{\alpha_1}{N}j\right)\psi_{B0}. \tag{S2-24}$$

The solution to Eq. (S2-24) is

$$\psi_{B0} = C\exp\left[-\sqrt{\frac{1}{2}}\frac{\sin\frac{K_\rho a}{2\sqrt{2}}}{1 + 2\cos\frac{K_\rho a}{2\sqrt{2}}}\left(\kappa_2 a j + \frac{\alpha_1}{2N}j^2\right)\right], \tag{S2-25}$$

where C is a normalization factor. The above analytic results for both eigenvalues and eigenvectors agree well with numerical calculations (Fig. S3).

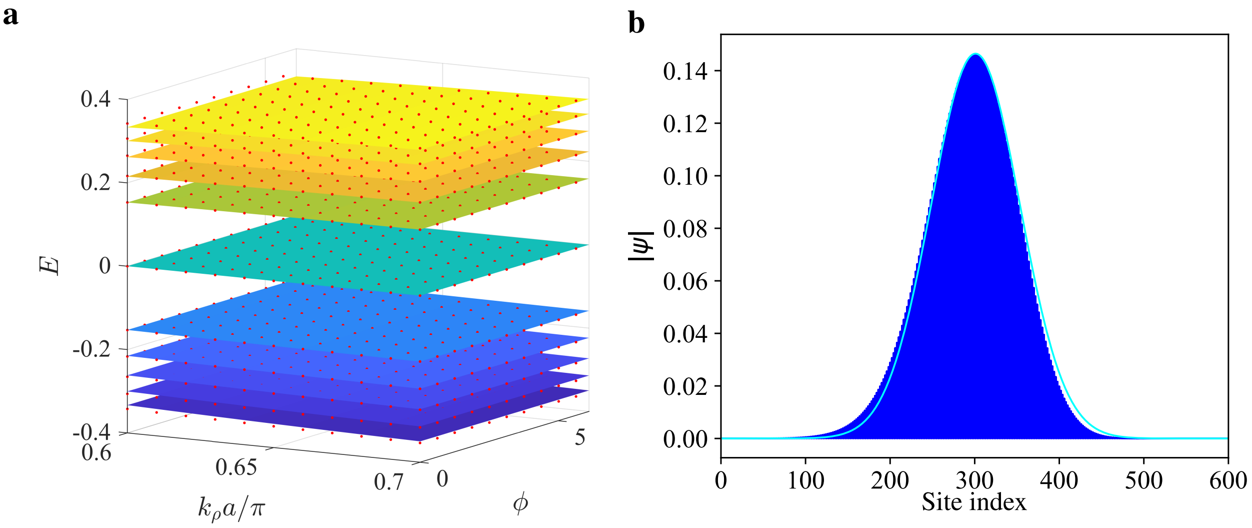

FIG. S3. **a**, The plot of band structure along k_ρ and ϕ directions. Here $k_\rho = \sqrt{k_1^2 + k_2^2}$, $N = 300$, $\alpha_1 = \pi$, $\alpha_2 = 0$. The color surface and red dots display numerical results and theoretical prediction Eq. (S2-16)(S2-19), respectively. **b**, Plot of the zeroth Landau level's wavefunction with $(k_1, k_2) = (0, \pi/2a)$. Parameters are the same as **a**. Blue and cyan lines are numerical results and theoretical prediction Eq. (S2-25), respectively.

D. Boundary modes

Nodal line semimetals can host topological boundary modes called drumhead surface states [1]. In our inhomogeneous nodal ring system, these drumhead surface states also exist and are smoothly connected to the zeroth LL as $k_{1,2}$ is swept (Fig. S4a). An interesting feature of the boundary modes is their surface-dependent property. Due to the system's inhomogeneity, the nodal rings near the bottom and the top surfaces have different radii. Consequently, the drumhead surface states spread different areas in the k_1–k_2 plane, according to the projection of the nodal ring in the neighboring bulk area (Fig. S4b).

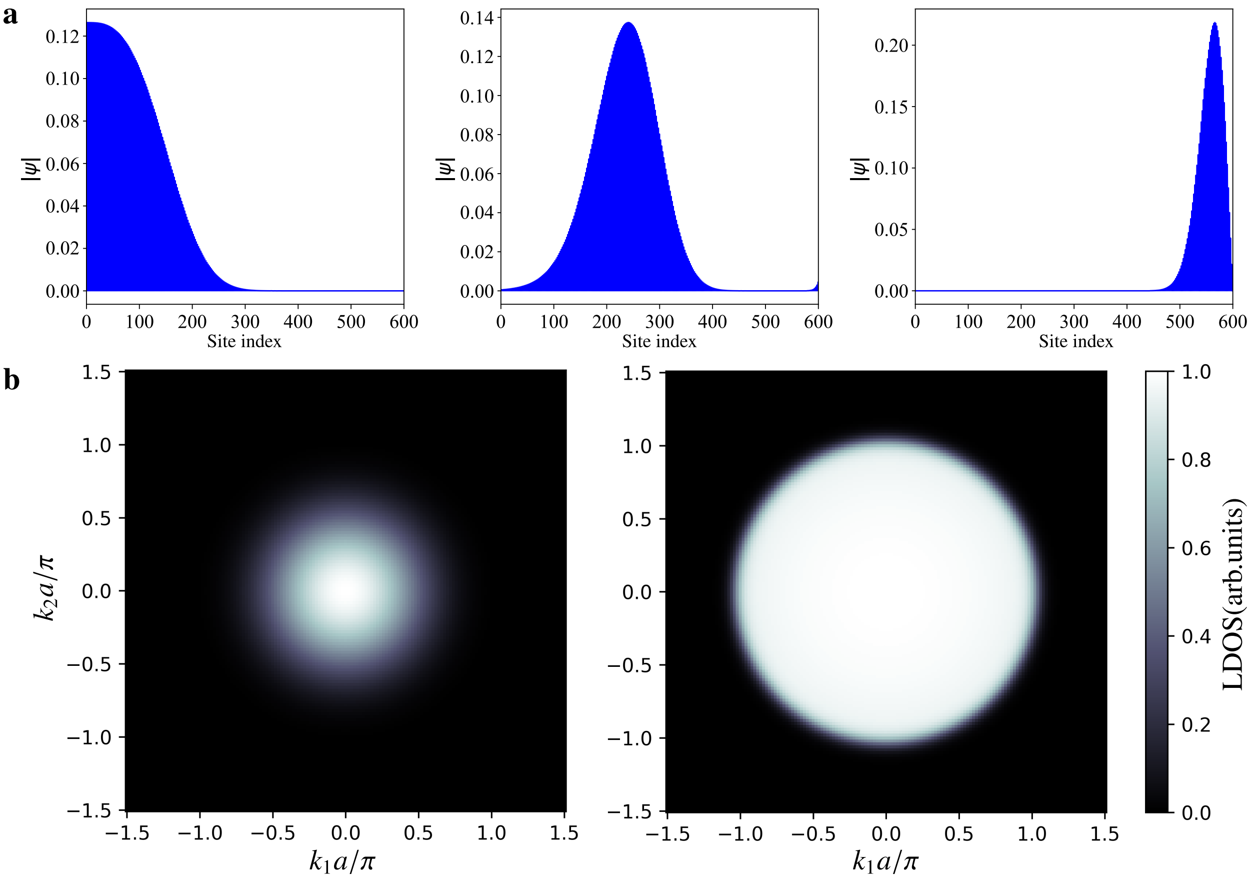

FIG. S4. **a**, Plots of the wavefunctions of the zeroth Landau level for different $(0, k_2)$. From left to right, $k_2a = 0$, 0.4π, π, respectively. **b**, Plots of the local density of states for the bottom surface and the top surface. Parameters: $N = 300, \alpha_1 = \pi, \alpha_2 = 0$.

III. ACOUSTIC STRUCTURE SIMULATION

In this section, we present more details on the simulation of the acoustic structure.

For different values ξ, Figure S5 shows that the nodal ring can be approximately regarded as a circle.

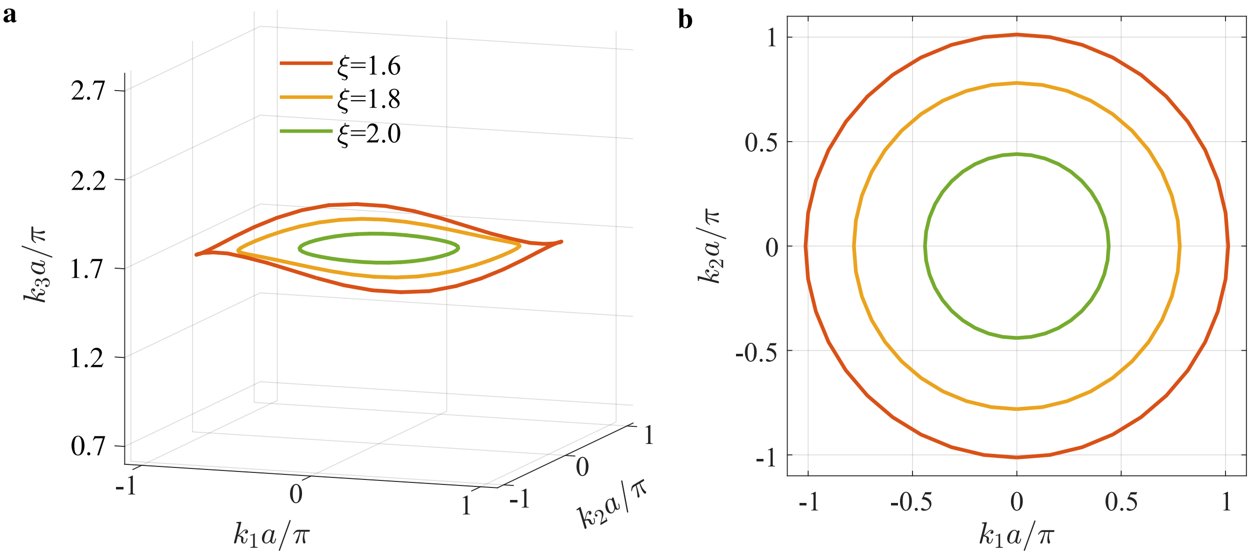

FIG. S5. **a**, Plot of the relationship between the nodal ring and ξ in the **k**-space. **b**, Plot of the relationship between the nodal ring and ξ in the $k_1 - k_2$ plane.

Figure 3e in the main text displays the LL along the k_2 direction when the radius of the nodal ring varies from $0.5\pi/a$ to $0.9\pi/a$ ($B = 0.4\frac{\sqrt{3}\pi}{Na^2}$). Here we provide bandstructure with different B. Figure S6a shows the band structure along k_2 direction for $\alpha_1 = 0.6\pi, \alpha_2 = 0.4\pi$, and the radius of nodal ring varies from $0.4\pi/a$ to π/a ($B = 0.6\frac{\sqrt{3}\pi}{Na^2}$). Figure S6b shows the band structure along k_2 direction for $\alpha_1 = 0, \alpha_2 = 0.7\pi$, and the radius of nodal ring is $0.7\pi/a$ ($B = 0$).

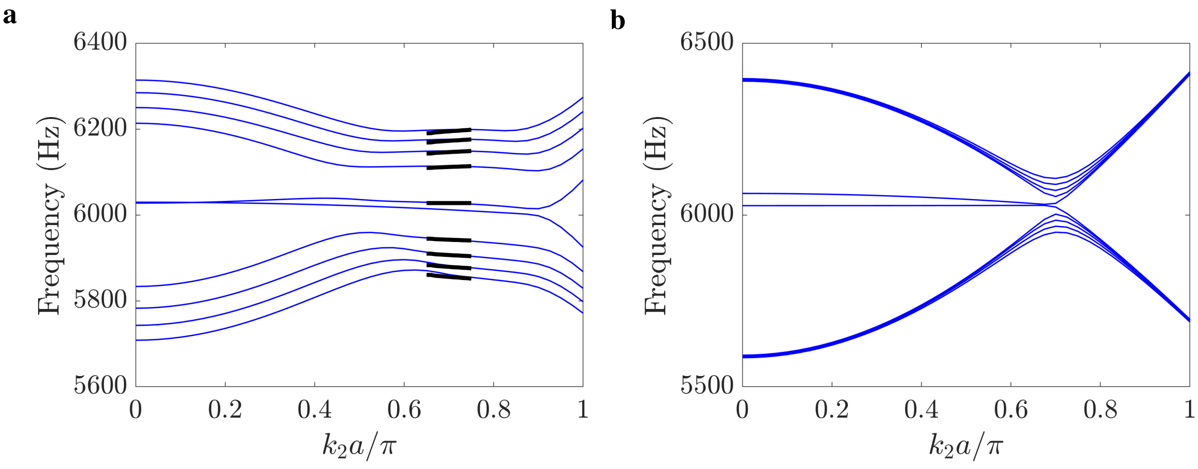

FIG. S6. Plot of band structure along k_2 direction. **a**, the radius of nodal ring varies from $0.4\pi/a$ to π/a ($B = 0.6\frac{\sqrt{3}\pi}{Na^2}$). **b**, the radius of nodal ring is $0.7\pi/a$ ($B = 0$).

Figure 3e in the main text displays the LL along the k_2 direction. To further prove the LLs are

almost flat in k space, we plot band structure in $k_\rho - \phi$ plane in Figure S7. Figure S7a shows the band structure for $\alpha_1 = 0.6\pi, \alpha_2 = 0.4\pi$, and the radius of nodal ring varies from $0.4\pi/a$ to π/a ($B = 0.6\frac{\sqrt{3}\pi}{Na^2}$). Figure S7b shows the band structure $\alpha_1 = 0.4\pi, \alpha_2 = 0.5\pi$, and the radius of nodal ring varies from $0.5\pi/a$ to $0.9\pi/a$ ($B = 0.4\frac{\sqrt{3}\pi}{Na^2}$).

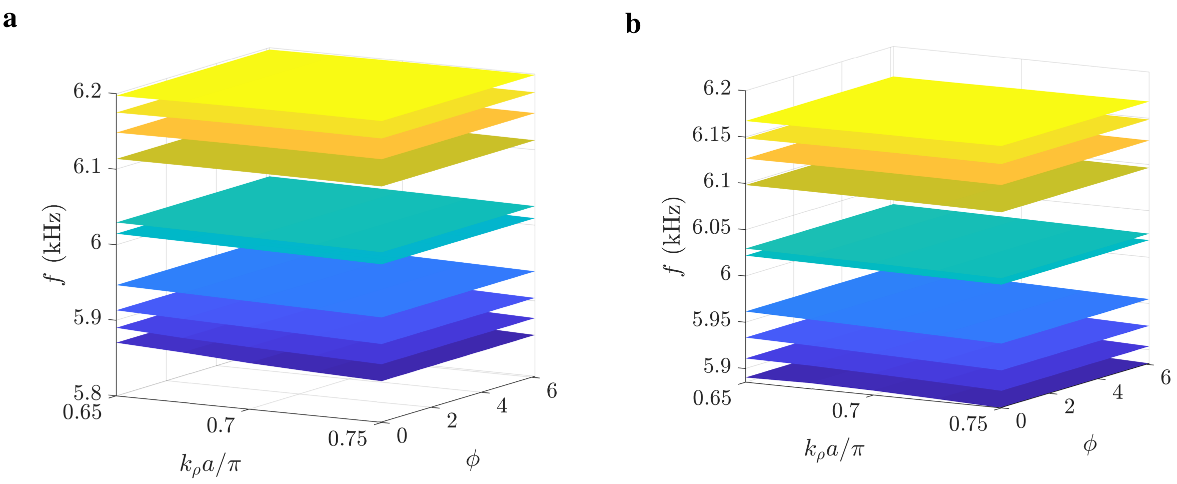

FIG. S7. Plot of band structure along k_ρ and ϕ directions. Here $k_\rho = \sqrt{k_1^2 + k_2^2}$, $N = 300$. The color surface displays simulation results. **a**, the radius of nodal ring varies from $0.4\pi/a$ to π/a ($B = 0.6\frac{\sqrt{3}\pi}{Na^2}$). **b**, the radius of nodal ring varies from $0.5\pi/a$ to $0.9\pi/a$ ($B = 0.4\frac{\sqrt{3}\pi}{Na^2}$).

Figure 4d in the main text represents the measured spectrum at the same bulk site, which shows peaks on $n = \{-1, 0, 1\}$ LLs. Its position is shown in Figure S8.

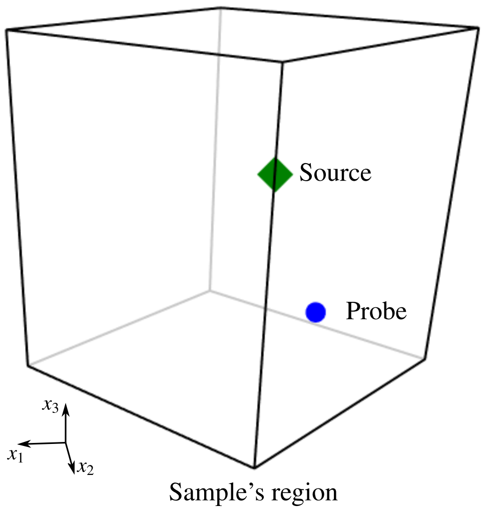

FIG. S8. Illustration of the position of the source (green marker) and detection position (blue marker).

IV. $k \cdot p$ EXPANSION IN ACOUSTIC STRUCTURE

This part provides the detailed derivation of the $k \cdot p$ expansion effective Hamiltonian in the acoustic system.

A. The Hamiltonian in the first quantization

The eigenvalue equation in the elastic wave is

$$-\nabla \cdot \left(\frac{1}{\rho(\mathbf{r})} \nabla p_{n\mathbf{k}}(\mathbf{r}) \right) = \frac{\omega_{n\mathbf{k}}^2}{v^2} \frac{1}{\rho(\mathbf{r})} p_{n\mathbf{k}}(\mathbf{r}), \tag{S4-1}$$

where $v = \sqrt{K(\mathbf{r}) / \rho(\mathbf{r})}$ is the sound speed. Here $\mathbf{k}$, n, ρ, and K are wavevector, band index, material density, and material stiffness, respectively. So Eq. (S4-1) can be written as

$$-\nabla \cdot \left(\frac{1}{\rho(\mathbf{r})} \nabla p_{n\mathbf{k}}(\mathbf{r}) \right) = \frac{\omega_{n\mathbf{k}}^2}{K(\mathbf{r})} p_{n\mathbf{k}}(\mathbf{r}). \tag{S4-2}$$

The wavefunction $p(\mathbf{r})$ has orthogonality and can be normalized as

$$\int_{\text{unit cell}} d\mathbf{r} p_{n\mathbf{k}}^*(\mathbf{r}) \frac{1}{K(\mathbf{r})} p_{n'\mathbf{k}}(\mathbf{r}) = \delta_{nn'}. \tag{S4-3}$$

The integration is over the unit cell without a statement for later convenience. We notice that $-\nabla \cdot \frac{1}{\rho(\mathbf{r})} \nabla$ is a Hermitian operator so that we can take the Hamiltonian as

$$H = -\nabla \cdot \frac{1}{\rho(\mathbf{r})} \nabla. \tag{S4-4}$$

Later, we will expand the Hamiltonian near $\mathbf{K}$, defining $\mathbf{k} = \mathbf{K} + \boldsymbol{\kappa}$. From the Bloch theory, we have

$$p_{n\mathbf{k}}(\mathbf{r}) = e^{i\mathbf{k}\cdot\mathbf{r}} u'_{n\mathbf{k}}(\mathbf{r}) = e^{i\boldsymbol{\kappa}\cdot\mathbf{r}} e^{i\mathbf{K}\cdot\mathbf{r}} u'_{n\mathbf{k}}(\mathbf{r}) = e^{i\boldsymbol{\kappa}\cdot\mathbf{r}} u_{n\mathbf{k}}(\mathbf{r}). \tag{S4-5}$$

Then

$$Hp_{n\mathbf{k}} = e^{i\boldsymbol{\kappa}\cdot\mathbf{r}} \rho^{-1} \boldsymbol{\kappa}^2 u_{n\mathbf{k}} - 2e^{i\boldsymbol{\kappa}\cdot\mathbf{r}} \rho^{-1} i\boldsymbol{\kappa} \cdot \nabla u_{n\mathbf{k}} - e^{i\boldsymbol{\kappa}\cdot\mathbf{r}} \left(\nabla \rho^{-1} \right) \cdot i\boldsymbol{\kappa} u_{n\mathbf{k}} - e^{i\boldsymbol{\kappa}\cdot\mathbf{r}} \nabla \cdot \left(\rho^{-1} \nabla u_{n\mathbf{k}} \right). \tag{S4-6}$$

$$\omega_{n\mathbf{k}}^2 K^{-1} p_{n\mathbf{k}} = \omega_{n\mathbf{k}}^2 K^{-1} e^{i\boldsymbol{\kappa}\cdot\mathbf{r}} u_{n\mathbf{k}}. \tag{S4-7}$$

From Eqs. (S4-1)(S4-6)(S4-7), we have

$$H(\mathbf{k}) u_{n\mathbf{k}} = \omega_{n\mathbf{k}}^2 K^{-1} u_{n\mathbf{k}}, \tag{S4-8}$$

$$H(\mathbf{k}) = H + H_{k\cdot p} + \rho^{-1} \boldsymbol{\kappa}^2, \tag{S4-9}$$

$$H_{k\cdot p} = \boldsymbol{\kappa} \cdot \boldsymbol{\pi}, \quad \boldsymbol{\pi} = -i \left(2\rho^{-1} \nabla + \left(\nabla \rho^{-1} \right) \right). \tag{S4-10}$$

We rewrite Eq. (S4-8) as the standard eigenvalue equation

$$H(\mathbf{k})\,u_{\mathbf{k}} = \omega_{\mathbf{k}}^2 K^{-1} u_{\mathbf{k}}. \tag{S4-11}$$

B. The Hamiltonian in the second quantization

From Eqs. (S4-3)(S4-5), we have

$$\int d\mathbf{r} u_{n\mathbf{k}}^{*}(\mathbf{r}) \frac{1}{K(\mathbf{r})} u_{n'\mathbf{k}}(\mathbf{r}) = \delta_{nn'}. \tag{S4-12}$$

As $\{u_{n\mathbf{K}}(\mathbf{r})\}$ is complete, we can expand eigenfunction $u_{n\mathbf{k}}(\mathbf{r})$ as

$$u_{n\mathbf{k}}(\mathbf{r}) = \sum\nolimits_{n'} A_{nn'}(\mathbf{k})\, u_{n'\mathbf{K}}(\mathbf{r}). \tag{S4-13}$$

So

$$H(\mathbf{k})\,u_{n\mathbf{k}} = \sum\nolimits_{n'} A_{nn'}(\mathbf{k}) \left(H + H_{k\cdot p} + \rho^{-1}\boldsymbol{\kappa}^2\right) u_{n'\mathbf{K}}(\mathbf{r}) = \sum\nolimits_{n'} A_{nn'}(\mathbf{k}) \left(\omega_{n'\mathbf{K}}^2 K^{-1} + \rho^{-1}\boldsymbol{\kappa}^2 + H_{k\cdot p}\right) u_{n'\mathbf{K}}(\mathbf{r}), \tag{S4-14}$$

$$\omega_{n\mathbf{k}}^2 K^{-1} u_{n\mathbf{k}} = \omega_{n\mathbf{k}}^2 K^{-1} \sum\nolimits_{n'} A_{nn'}(\mathbf{k})\, u_{n'\mathbf{K}}(\mathbf{r}). \tag{S4-15}$$

From Eqs. (S4-8)(S4-14)(S4-15), we have

$$\sum\nolimits_{n'} A_{ln'}(\mathbf{k}) \left(\omega_{n'\mathbf{K}}^2 K^{-1} + \rho^{-1}\boldsymbol{\kappa}^2 + H_{k\cdot p}\right) u_{n'\mathbf{K}} = \omega_{l\mathbf{k}}^2 K^{-1} \sum\nolimits_{n'} A_{ln'}(\mathbf{k})\, u_{n'\mathbf{K}}.$$

Here, we replace n with l for later convenience. Left multiplied $u_{n\mathbf{K}}^{*}$ and integrated over the unit cell, we get

$$\sum\nolimits_{n'} \left(\omega_{n\mathbf{K}}^2 \delta_{nn'} + \overline{V^2}_{nn'}\boldsymbol{\kappa}^2 + \boldsymbol{\kappa}\cdot\boldsymbol{\pi}_{nn'}\right) A_{ln'}(\mathbf{k}) = \omega_{l\mathbf{k}}^2 A_{ln}(\mathbf{k}). \tag{S4-16}$$

where

$$\overline{V^2}_{nn'} = \int d\mathbf{r} u_{n\mathbf{K}}^{*} \rho^{-1} u_{n'\mathbf{K}}, \tag{S4-17}$$

$$\begin{aligned} \boldsymbol{\pi}_{nn'} &= -i \int d\mathbf{r} u_{n\mathbf{K}}^{*} \left(2\rho^{-1}\nabla u_{n'\mathbf{K}} + \left(\nabla\rho^{-1}\right) u_{n'\mathbf{K}}\right) \\ &= -i \int d\mathbf{r} \left(\rho^{-1}\left(u_{n\mathbf{K}}^{*}\nabla u_{n'\mathbf{K}} - (\nabla u_{n\mathbf{K}}^{*})\, u_{n'\mathbf{K}}\right) + \nabla\left(\rho^{-1} u_{n\mathbf{K}}^{*} u_{n'\mathbf{K}}\right)\right). \end{aligned} \tag{S4-18}$$

In fact, Eqs. (S4-16)(S4-17)(S4-18) tell us the second quantized Hamiltonian

$$\mathcal{H}_{nn'} = \omega_{n\mathbf{K}}^2 \delta_{nn'} + \overline{V^2}_{nn'}\boldsymbol{\kappa}^2 + \boldsymbol{\kappa}\cdot\boldsymbol{\pi}_{nn'}, \tag{S4-19}$$

and eigenvalue equation

$$\mathcal{H}A = \omega_{\mathbf{k}}^2 A, \tag{S4-20}$$

with

$$A = (A_1, A_2, \cdots)^T . \tag{S4-21}$$

Until now, all of our derivations are based on the wave equation Eq. (S4-1), and no approximation is assumed. As we are interested in the lowest two bands and they are according to the lowest order eigenmodes, it is a good approximation that

$$\boldsymbol{\pi}_{nn'} \approx 0 \quad \text{if } n, n' \neq 1, 2. \tag{S4-22}$$

We only keep linear terms; then we get the reduced Hamiltonian

$$\mathcal{H}_{nn'} = \omega_{n\mathbf{K}}^2 \delta_{nn'} + \boldsymbol{\kappa} \cdot \boldsymbol{\pi}_{nn'}, \quad n, n' = 1, 2. \tag{S4-23}$$

with $\boldsymbol{\pi}_{nn'} = \left(\pi_{nn'}^{(1)}, \pi_{nn'}^{(2)}, \pi_{nn'}^{(2)}\right)$ in $O - u_1 u_2 u_3$ coordinate system. We do the $k \cdot p$ expansion near the degenerate point $\mathbf{K} = (0, K_2, 0)$, so $\omega_{1\mathbf{K}} = \omega_{2\mathbf{K}} = \omega_{\mathbf{K}}$. Numerical calculation shows that Eq. (S4-23) has simplified form

$$\mathcal{H} = \omega_{\mathbf{K}}^2 \sigma_0 + \sigma_2 \left(v_{21}\kappa_1 + v_{22}\kappa_2 + v_{23}\kappa_3\right) + \sigma_3 \pi_{11}^{(2)} \kappa_2, \tag{S4-24}$$

with components list in Supplementary Table. I.

Supplementary Table I. $\boldsymbol{\pi}$ in $k \cdot p$ expansion, unit: v^2/a, $v = 346$m/s, $a = 40$mm.

$k_2 a/\pi$	$\pi_{11}^{(2)}$	v_{21}	v_{22}	v_{23}
0.65	-2.047712	-0.454526	0.201639	-6.140447
0.66	-2.075291	-0.468460	0.244487	-6.080075
0.67	-2.115545	-0.484657	-0.070947	-6.096307
0.68	-2.144671	-0.496069	-0.080528	-6.070662
0.69	-2.172647	-0.513779	0.057166	-6.060799
0.70	-2.200881	-0.526397	-0.074209	-6.037328
0.71	-2.226346	-0.543854	0.033169	-6.035195
0.72	-2.249296	-0.553188	-0.056590	-6.003315
0.73	-2.282225	-0.565868	-0.022637	-5.991396
0.74	-2.309043	-0.588370	0.066501	-5.956318
0.75	-2.343372	-0.602997	-0.017102	-5.932566

After a similar transformation, Eq. (S4-24) has the form

$$\exp\left[-i\frac{\pi}{4}\sigma_2\right]\mathcal{H}\exp\left[i\frac{\pi}{4}\sigma_2\right] = \omega_{\mathbf{K}}^2\sigma_0 + \sigma_1\pi_{11}^{(2)}\kappa_2 + \sigma_2\left(v_{21}\kappa_1 + v_{22}\kappa_2 + v_{23}\kappa_3\right). \tag{S4-25}$$

Now we have the two-band eigenvalue equation

$$\mathcal{H}'A' = \omega_{\mathbf{k}}^2 A', \tag{S4-26}$$

$$\mathcal{H}' = \exp\left[-i\frac{\pi}{4}\sigma_2\right]\mathcal{H}\exp\left[i\frac{\pi}{4}\sigma_2\right], \tag{S4-27}$$

$$A' = \exp\left[-i\frac{\pi}{4}\sigma_2\right]A, \quad A = [A_1, A_2]^T. \tag{S4-28}$$

C. Acoustic Landau levels based on $k \cdot p$ Hamiltonian

Similar to the tight-binding model, we show the consequences of the PMF. After we implement the vector field **A**, the effective Hamiltonian Eq. (S4-25) becomes

$$\mathcal{H} = \omega_{\mathbf{K}}^2\sigma_0 + h_1\sigma_1 + h_2\sigma_2, \tag{S4-29}$$

$$h_1 = \pi_{11}^{(2)}\left(\kappa_2 + A_2\right), \tag{S4-30}$$

$$h_2 = v_{21}\left(\kappa_1 + A_1\right) + v_{22}\left(\kappa_2 + A_2\right) + v_{23}\left(-i\partial_3 + A_3\right). \tag{S4-31}$$

$A_{1,2}$ is given by Eq. (S2-9). Then

$$2i\left[h_2, h_1\right] = \frac{2\sqrt{3}}{a^2}\frac{\alpha_1}{N}v_{23}\pi_{11}^{(2)}. \tag{S4-32}$$

From Supplementary Table. I, we have the fit result

$$v_{23}\pi_{11}^{(2)}\frac{a^2}{v^4} = -30.9159\left(\frac{k_2 a}{\pi}\right)^2 + 56.6909\left(\frac{k_2 a}{\pi}\right) - 11.2464. \tag{S4-33}$$

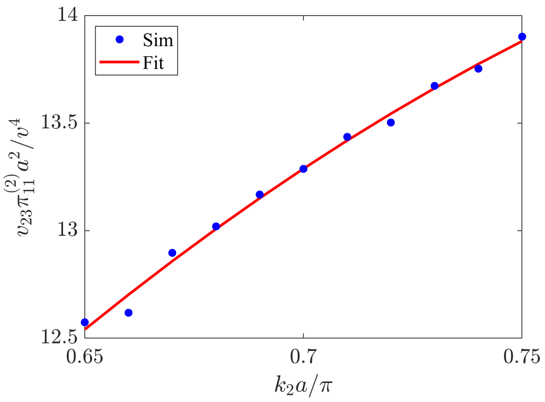

FIG. S9. Illustration of the relationship between $v_{23}\pi_{11}^{(2)}a^2/v^4$ and k_2a/π. The blue dots and red line are plotted based on Supplementary Table. I and Eq. (S4-33), respectively.

Similar to the derivation in the tight-binding model, the acoustic Landal levels are given by

$$\omega_n^2 = \omega_0^2 + \mathrm{sgn}\,(n)\ \sqrt{|n|\,|2i\,[h_2, h_1]|} \tag{S4-34}$$

From Eqs. (S4-32)(S4-34), we have

$$\omega_n = \sqrt{\omega_0^2 + \mathrm{sgn}\,(n)\left(\frac{v}{a}\right)^2 \sqrt{|n|\,2\sqrt{3}\frac{\alpha_1}{N}\left[-30.9159\left(\frac{k_2 a}{\pi}\right)^2 + 56.6909\left(\frac{k_2 a}{\pi}\right) - 11.2464\right]}}. \tag{S4-35}$$

Considering that the degenerate points form a nodal ring, we can approximate that

$$\omega_n = \sqrt{\omega_0^2 + \mathrm{sgn}\,(n)\left(\frac{v}{a}\right)^2 \sqrt{|n|\,2\sqrt{3}\frac{\alpha_1}{N}\left[-30.9159\left(\frac{ka}{\pi}\right)^2 + 56.6909\left(\frac{ka}{\pi}\right) - 11.2464\right]}}, \tag{S4-36}$$

with $k = \sqrt{k_1^2 + k_2^2}$.

V. SAMPLE PHOTOS

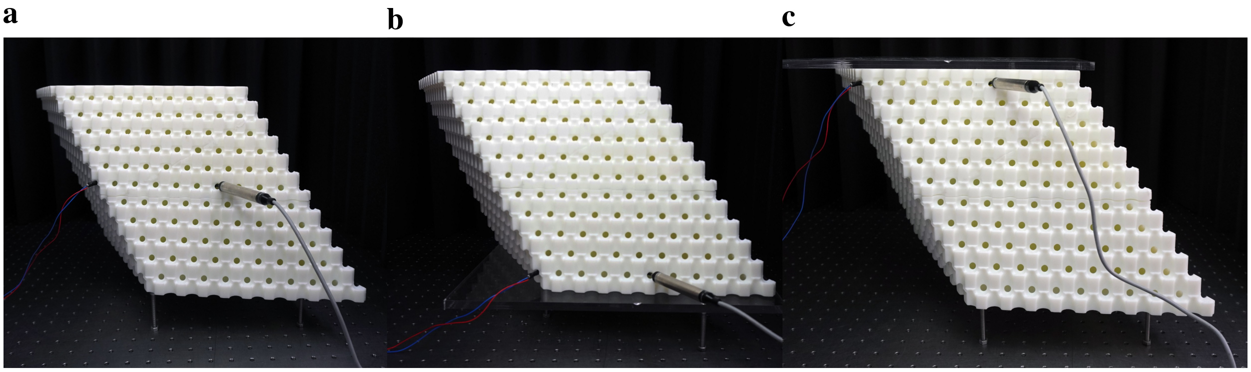

FIG. S10. **a**, Photo of the setup when we measure the LLs, the bottom and top surfaces are open. **b(c)**, Photo of the setup when we measure the bottom (top) drumhead surface state, an acrylic plate is applied at the bottom (top) surface.

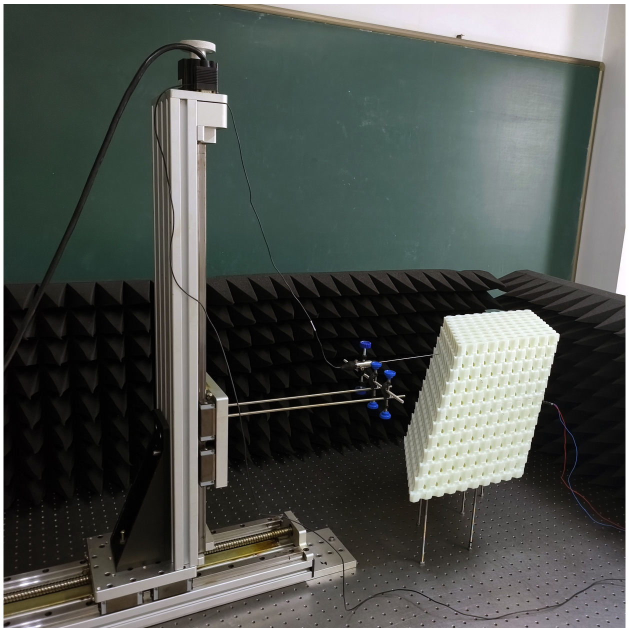

FIG. S11. The experiment setup. The source is inserted into the center of the sample. The probe is assembled on the mechanical arm. The arm is controlled by the stepping motor, allowing precise field mapping.

VI. SUPPLEMENTARY REFERENCES

[1] C. Fang, H. Weng, X. Dai, and Z. Fang, Topological nodal line semimetals, Chin. Phys. B **25**, 117106 (2016).
